# Frail Older Persons’ Experiences of Information and Participation in Hospital Care

**DOI:** 10.3390/ijerph16162829

**Published:** 2019-08-08

**Authors:** Berit Forsman, Ann Svensson

**Affiliations:** 1 Department of Health Sciences, University West, 461 86 Trollhättan, Sweden; 2 School of Business, Economics and IT, University West, 461 86 Trollhättan, Sweden

**Keywords:** communication, frail older persons, health literacy, information, person-centered care (PCC)

## Abstract

The aim of this paper is to describe frail older persons’ experiences of hospital care of information and participation when being an inpatient at a hospital. A qualitative method was used. Data were collected at the hospital from 20 interviews with frail older patients, together with observations in the environment at the hospital ward. A content analysis was performed. Patients experienced not receiving information about their care and rehabilitation, or receiving such information in noisy surroundings. They experienced situations of misunderstanding related to their medication, which indicates the need for appropriate discharge calls for frail older patients. They expressed feelings of distress concerning the future, caused by hasty admissions or relatives’ problems to handle the situation. The results highlight the need to receive appropriate information and to participate in decision-making. The level of health literacy should be taken notice of when giving information, using peaceful and quiet environments when informing frail older persons. Person-centered care should be recognized to a greater extent in order for healthcare professionals to give information to frail older people in a health literacy-friendly way. This might make it easier for frail older persons to participate in a partnership in care.

## 1. Introduction

Frail older persons are described as multi-diseased and vulnerable patients in need of medication and hospital care, and as the population within the Western world is growing older, this population group poses medical challenges for society of today and of the future [1]. These persons require healthcare from several care-givers within the health services, including hospital-based acute care services, primary care services, and community care services. Moreover, many representatives of this patient group are readmitted within three months [2]. Many older persons wish, despite chronic illnesses, to remain independent and thus, wish to live in their own homes and to take care of themselves for as long as possible [3,4]. 

Studies show that frail older persons are often maltreated, as healthcare services are faced with insufficient coordination and unclear distribution of responsibilities between different caregivers [5,6]. Nurses also describe a need for improved communication and coordination during transition of older people between nursing homes and hospitals [7]. Lack of coordination of care and ineffective discharge planning can lead to decreased patient satisfaction, and a higher number of hospital readmissions due to complications [8]. According to the Swedish Patient Safety Act, the legal right to information and influence is crucial for the patient´s autonomy. Patients should receive accurate and personalized information in order to be able to participate in their own care, as well as being a partner in the healthcare process. Healthcare professionals should be open and responsive to each individual, perceiving the patient as an expert, and then treat the patient as a partner and an equal person. This person-centered care (PCC) is proven to have a positive impact on healthcare outcomes [9,10]. Moreover, frail older patients might require an environment that is peaceful and silent, and which lacks too many distractions, in order to understand information about their own health (Swedish Agency for Health Technology Assessment and Assessment of Social services). A basis for patients participating in their own healthcare is that the overall conditions are supporting their understanding and comprehension of information concerning their own health [11]. Therefore, a better understanding of frail older persons’ experiences of their person-centered hospital care, with special interest in information and participation, are of vital importance. Thus, the aim of this paper is to describe frail older persons’ experiences of hospital care of information and participation when being an inpatient at a hospital. We end this paper by discussing our findings for implications on how PCC can be better supported by healthcare professionals and how information can be given to frail older persons in a more health literacy-friendly way. 

### 1.1. Insufficient Communication

Frail older persons tend to meet many different physicians in the specialist hospital departments where they are treated for various complex diseases by specialized physicians, and test results are not always confirmed [12]. Communication between hospital doctors and the primary care doctor responsible for a specific patient’s medication is often non-existent [13], and subsequently, the medication is not adjusted to the patient’s actual situation. Hospital physicians do not always take time to inform their patients about their treatments and medicines [6,14], or about changes regarding these treatments and medicines, which might lead to extensive medication errors. As a consequence, medicines prescribed during a hospital stay might still be on that patient’s medicine list in the form of someone else’s responsibility. The primary care physicians, however, wish to be involved to a greater extent when their patients are admitted to hospital [15,16]. Insufficient continuity in the transition between hospital care and primary care has been identified as the main cause of medical mistakes. Such medical mistakes may also increase the need for hospital readmission within three months [14,17]. Poor quality in medication prescriptions has also been proven to be directly linked to poor life quality within the group of frail older persons [18]. Insufficient communication between hospitals and primary care services may lead to maltreatment, unnecessary hospital care, and readmission of frail older persons, which is a world-wide phenomenon within healthcare [12,17,19]. Obstacles have been identified for obtaining effective communication and care planning, from a professionals’ point of view. There exist opportunities to increase communication with patients and relatives, and improve collaboration between care-givers [6].

### 1.2. Health Literacy

The concept of health literacy is described as a heterogeneous phenomenon by which individuals can understand and comprehend information concerning their own health [11]. According to Sykes et al. [20], health literacy is a concept which can be defined in two ways. The first definition describes the concept as polarizing, in that functional abilities like an individual’s reading comprehension and language are of importance. Health literacy can therefore be defined based on an evaluation of an individual’s abilities as high or low. The second way of describing the phenomenon is more complex. It comprises a broader type of decision-making and varies between individuals depending on their context. Political governance is a means of improving health literacy based on the individual context towards more equal terms within healthcare and a better utilization of the healthcare system [20]. Sorensen et al. [21] identified several dimensions of health literacy, referring to competencies related to accessing, understanding, appraising, and applying health information. Within European public health research, health literacy “encompasses people’s knowledge, motivation and competences to access, understand, appraise, and apply health information in order to make judgments and take decisions in everyday life concerning healthcare, disease prevention and health promotion to maintain or improve quality of life during the life course”, as defined by Visscher et al. [22].

Increased participation in care-related decisions contributes to fewer readmissions and reduces anxiety in frail older persons [23]. According to Dennison et al. [24], adequate health literacy is associated with sufficient knowledge about heart failure and higher confidence in patients’ self-care. Persons with inadequate health literacy run an increased risk of negative outcomes, such as hospital readmission. Patients in Ekdahl et al.’s study [23] did not understand the medical language, which made it impossible for them to take part in medical decisions. Health literacy is an area which has an impact on the communication between healthcare professionals and their patients. Using models to individualize cues and to modify the communication between older persons and care-givers can be helpful for empowering patients to acquire better health [25].

### 1.3. Person-Centered Care

The person-centered care (PCC) approach to nursing assumes a more holistic care and contains both cultural and contextual challenges [26]. Morgan and Yoder [27] describe the development of the concept, from the 1940s first being focused on the patient, and during the late 1980s to the person given care. Dimensions like care regardless of the healthcare setting, with respect for the patient’s values, preferences, and expressed needs, coordination, emotional support, involvement of friends and family, transition, and continuity. PPC should not only occur between a care provider and a patient, but also on an organizational level. Professionals can perform PCC by using their expertise while developing an openness and responsiveness to an individual’s life-world [26]. In the 2000s, there is a statutory requirement for well-functioning healthcare to meet individual needs [28]. PCC might generate increased feelings of well-being and good health, and also more satisfied persons. A strategy to make this possible is to provide nurses and doctors with adequate resources to give medical information and perform discharge calls. A change in the approach towards PCC implies a shift of roles, as health professionals, as a consequence, have to perceive the patient as an expert, rather than the other way around [28,29]. A person in need of care should be treated as a partner and an equal. By listening to the person’s story and identifying his or her possibilities and resources, a foundation for a partnership in care can be achieved [30,31]. PCC also requires conscious ethics and a relationship based on mutual respect and understanding for a person’s self-esteem and desires [23,29,32]. 

The previously presented studies describe the issue from the perspective of healthcare professionals focused on organizational problems. There are, however, a few studies focusing on the perspective of frail older persons. The patients’ transitions to their homes is experienced as unsafe and troublesome, especially for frailer participants [33]. Moreover, findings from Kirsebom et al. [7] indicate that 16% of the readmissions and transitions from nursing homes to hospital could have been avoided. The reason for this is to be investigated further. There is a challenge which involves increasing the participation of frail older patients in medical decision-making [23]. Due to functional disabilities related to hearing and memory, not many studies have been conducted that focus on the perspective of frail older persons. The objective of this study is to describe and capture experiences of information and participation from persons who are directly affected by hospital healthcare and its consequences. This paper is thus emphasizing the experiences of the frail older persons at hospitals.

## 2. Method 

This study has a qualitative approach in order to deepen the understanding for frail older persons’ experiences of their hospital care, with special interest in information and participation. A qualitative approach is particularly suitable for studies conducted within complex healthcare services in which good quality is measured not only according to, for example, waiting time at an emergency department, but also by an understanding of the experience of waiting time [34]. The nature of the clinical environment, the adequacy of communication in relation to health professionals and the context and manner in which treatment is delivered, are other important areas of interest. The qualitative method used deepens the understanding for the informant´s experiences related to the objective of the paper [35]. The study was set within the Swedish healthcare sector at a public urban hospital in the western part of Sweden. This is the main hospital, the only one with emergency healthcare, and serves about 300,000 inhabitants in this geographical area. This hospital treats all patients free of charge, regardless of the patient’s incomes or insurances. All patients were readmitted to the very same hospital where the study was conducted.

### 2.1. Participants

For this study, purposive sampling was used. All participants included were patients between 65 to 85 years of age who had been readmitted to the hospital care within three months, due to diagnoses such as chronic obstructive pulmonary disease (COPD), asthma, hypertonia, heart failure, or ulcers. Patients without cognitive disorders were chosen. As the included patients should also be close to being discharged from the hospital, we tried to achieve the highest possible validity and reliability of the study [36]. A total of 26 patients who met the criteria were identified; however, four of these patients were either too tired or did not want to participate in the study. All included patients were frail but still able to both hear and understand the questions asked during the interview. There were not any hindrances to this study in the form of memory or hearing disabilities of the patients.

Twelve women and eight men were included as participants, where 19 of the participants were living in their own homes and one was living in special accommodation provided by the municipality. Most of the participants received some help from relatives or from the municipality’s social care service. 

The patients were contacted on the very same day the interviews were to be conducted, and they received information in written form about the study and their right to withdraw from participation at any time without consequences. 

### 2.2. Data Collection

Qualitative data was gathered from 20 informants by using a semi-structured interview guide, designed to be used for in-depth interviews. The interviews addressed the informants’ experiences and perceptions of the care they had received during and in between readmissions to the hospital, with a special interest in information and participation. The following themes were asked about at the interviews:

- How it has been at home since discharge;
- Experience of healthcare during last admission;
- Participation in planning for discharge from last admission;
- Information at last discharge;
- Contacts to get more information;
- Contacts with home care and primary care;
- Participation and contacts for readmission;
- Information about medication and its changes; and
- Planning for this discharge, and the home stay.

Both authors were present at the interviews; one conducted the interviews and one gave information to the patients and recorded the interviews. The authors were both responsible for making the frail older persons feel comfortable and for giving them sufficient time to describe their experiences. The interviewer has, during many years, worked as a registered nurse, and sometimes still does that, thus being well acquainted with the environment at the hospital’s wards. The interviews were performed at the hospital where the frail older patients were receiving their care. The settings were acute medical care wards, where noisy surroundings, as well as interruptions from for example, fellow patients or professionals, could be heard on the recordings. The situation therefore made it possible to observe the context of the surroundings and the context where communication between healthcare professionals and patients takes place [37]. Visual observation of participation and personalized information given was also notified during the interviews. All interviews were audiotaped and transcribed verbatim. Every interview lasted for approximately 14 min.

### 2.3. Data Analysis

The data analyzed in this study consist of transcribed text from the interviews. The qualitative data was structured and analyzed by the use of qualitative content analysis [35,38,39]. The analysis was conducted by both of the interviewers, and also discussed several times between the authors who were participating at the interviews. The units of analysis in this study consisted of segments of text from interviews about frail older patients’ experiences and perceptions of their hospital, care with special interest in information and participation. The transcribed texts were read through several times in order to obtain a sense of the wholeness. The texts were then divided into meaningful units that were condensed and labeled into a code. During this process, it was important not to lose sight of the textual and narrative structure of the qualitative data, and to pay attention to the context of the data items [38]. 

Examples of meaningful units, condensed meanings, and codes are shown in Table 1.

The various codes were compared based on differences and similarities, and then organized into three categories. The categories are (1) sufficiency of information and coordination, (2) varying of discharge routines, and (3) feelings concerning the future.

Observational data were analyzed by re-listening on the tape-recordings with focus on the surroundings of the interview situations. This analysis gave evidence of the noisy context where the frail older patients often receive their care at a hospital ward. The analysis of the observational data was mixed with the content analysis of the interviews. As in the cases P15, P19, and P1, the data from the interviews were confirmed by the authors’ observations. The authors noticed nice, calm, and friendly surrounding, as well as noisy and overcrowded wards.

### 2.4. Ethical Considerations

The data has been treated confidentially and quotes from the interviews have been shortened and formulated so as to provide anonymity. The participants were given both written and oral information about the study, and informed written consent was obtained from all participants prior to their participation, in accordance with the Declaration of Helsinki, 2008. This study has been approved by the Ethical Committee at University West (Dnr: 2014/458 B22).

## 3. Findings

The findings of the study, a content analysis based on frail older patients’ experiences and perceptions of hospital care with special interest in information and participation, is presented in three categories. In cases in which patients experienced and/or perceived positive experiences related to the category, it is presented at the beginning of each paragraph. Undesirable perceptions or experiences, on the other hand, are presented at the end of the paragraph. The findings of the content analysis are illustrated with citations from the transcribed interviews. 

### 3.1. Sufficiency of Information and Coordination

There were five informants who expressed feelings and perceptions of being well-informed during their period in hospital care. Patients who felt well-informed considered themselves to have a sufficient understanding of their medication, as well as the given care. One patient, who had been readmitted due to breathing problems described how the nurses and doctors gave him medications and “dried him out properly”, which helped him recover from his illness. Another patient described feelings of being protected from the risk of having another minor stroke by the doctor’s prescription of Warfarin. A third patient had been given some written information to read through before the beginning of the medical treatment. The doctors wanted to try a new medication, which was a drug that could make heart failure patients feel better by making the blood go out to all stations. The patient had a room of her own and a doctor also came to inform her during our interview. The authors noticed a nice, calm, and friendly surrounding and she appreciated the team’s concern for her, as illustrated by the following comment:

“They are marvelous, I mean, I am 75 years old.”

(P15)

Another patient reported feeling contented with the given care during the first week after discharge, but he soon started to feel worse again, which was probably due to concerns regarding the lack of information about the date and time for planned appointments at the primary care center. This patient was told that he would have to wait for three months, which made him worried and later caused him to call for an ambulance.

“It took so long before I could get any help from the primary care center—they did not send me a medical appointment time until in the middle of the following month.”

(P13)

Other informants were not satisfied at all with the given information and the coordination of the given hospital care, or they believed they had been sent home too early. One patient reported wanting to undergo more examinations and not understanding why his blood-pressure medicine had been removed. He wanted to stay in the hospital for a couple of days in order to check his blood pressure daily, as he knew that if it got high again, he would have to return to hospital once again, which he considered unnecessary and inconvenient. Another patient felt insecure because planned X-ray tests had not been carried out for a reason he did not understand or had not received information about. When he asked the nurses about this, they said the doctor was gone for the day, and the doctor on call did not know anything about his case. Some patients described experiences of not being informed at all, or being informed about the given care and rehabilitation at home in stressful and noisy surroundings. This can also be confirmed by the authors’ observations of the noisy and sometimes stressful surroundings which constitute the patient context, which is illustrated by the following comment:

“I got some information, but it was so hastily done on a late Sunday night. They had a lot to do, but I may well blame myself for not remembering what they said.”

(P19)

There were also several patients who did not know, or were not completely informed about, what drugs had been prescribed or removed, or whether the dose of their medication had been changed or not. One patient (P14) felt sure she had been prescribed a drug to help her stop vomiting, and she thought that the doctors had also added another medication that very same morning but was not sure about it. 

Two other patients commented on their lack of knowledge about what medication they were prescribed. They knew the names and indications of some of the drugs, but far from all of them. This experience led to uncertainty and can be illustrated by the following comment:

“I know some, I do, but, there are many different tablets… a diuretic, an antitrombotic… and Panadol, Ergenyl?… don’t ask me all their names.”

(P20)

The patient had not yet been offered a (documented) drug review.

Another patient claims about the healthcare performance:

“No, I wanted them to do a bit more follow-up work on my blood pressure.”

(P2)

Themes concerning improvements needed, according to sufficiency of information and coordination, can be summarized as follows:

- Unclear information about the obtained or given care;
- Not understanding or noticing changes in medication; and
- Information was given to hastily or in noisy surroundings.

### 3.2. Varying of Discharge Routines 

Six patients felt content with the discharge routines. Two patients had been given a care plan to store at home that held all necessary information that could be of use for either ambulance staff or primary care nurses working in the patient’s home. One informant received written information (on actual medication and on how to stay active) in her hand when she was discharged from hospital. The planning for discharge took place at the ward, and the information was given by a doctor and/or a nurse, as illustrated by the patients:

“They called after me and the whole team and made up plans for my discharge, so I have nothing to complain about at all.”

(P13)

The majority of the informants, however, described varying degrees of dissatisfaction with their hospital discharge. In some cases, the information was non-existent or very brief. One patient said he had only received a prescription and some tablets to take with him, and another one said the conversation had only lasted for a couple of minutes. One patient, who was discharged late at night, said that the conversation with the staff lasted for only a short time, and another informant said that he was already on his way out when they came to inform him. Vague or short discharge calls created feelings of insecurity. A comment that illustrates these experiences is:

“No, I wondered why the doctor didn´t come to give me a little bit of information, to sign me out, but I just went home.”

(P17)

Some patients did not know if they had had a formal discharge call or what category of a professional they had talked to. One patient described feelings of having had the opportunity to have a proper talk with the doctors and nurses, but that it did not turn out as expected. Another patient described the feeling that he or she might have had the possibility to talk, and a third one felt that it could have ended up as a good talk (discharge call), but that the patient did not dare to speak out and express his thoughts. At the end of the day, too many people had been involved and/or there had been no continuity, which both led to insecurity.

“A big crowd of people came to talk to me, and then I did not even know who the doctor was … It’s better when you have one person to talk to, when they are too many you don´t know where to start?”

(P1)

Themes concerning improvements needed according to varying of discharge routines can be summarized as follows:

- Being sent back home too early;
- No discharge call;
- Too many people involved at the same time; and
- No continuity.

### 3.3. Feelings Concerning the Future

There were patients who described their hospital stays as very positive experiences, as they had had staff and other patients to talk to. Some even worried about going back to their homes, since they knew they would feel lonely at home and not have anyone to talk to, except for some relatives and friends. One informant expressed the experience of professionals working for the home care services as having less time nowadays to sit down and just be a human being. Another patient said she did not feel content with her life due to her functional losses after a stroke which meant she could not ride her horses anymore. The patients who described feelings of distress concerning their future and how they would cope with their chronic diseases wished to remain living in their homes, but this depended to a great extent on how their illnesses would affect them.

“It depends on the illness, sometimes it just makes you stop… and you become totally exhausted.”

(P10)

Some patients had, in their own opinion, been discharged too early and/or when they were still in a bad physical condition. One patient was sent home only a couple of days after Christmas and had not been well in between his admissions. He was never in great shape, but suspected he must have had some kind of infection. Other informants perceived their relatives or next-of-kins as too weak to handle the situation and unable to cope with all practical matters. The upcoming situations had reduced their well-being and their satisfaction with their daily lives. There were also patients who described being unsatisfied with the help they received from the primary care services and/or the health services in their homes. Some informants felt they needed more help, which can be illustrated by the following comment:

“Home care is not working well, you see, I’m not afraid to tell you that they are always somewhere else … and they don’t hurry up when you need them.”

(P11)

An overall problematic statement was that many patients worried about their future, they felt dissatisfied and unsafe. This illustrates that the healthcare service is missing out on giving the patients information to involve them sufficiently enough in the care for them to apply health information to feel content of their lives.

Themes concerning improvements needed according to feelings concerning the future can be summarized as follows:

- Need for more care or health services;
- Concerns about inability to handle the need for daily care;
- No one to talk to/loneliness; and
- Sense of not being content with the life.

## 4. Discussion 

Frail older patients comprise a patient group with multiple chronic diseases and a great need of care, among whom some patients, but far from the majority, have been treated in accordance with their legal rights (Swedish Code of Statues from 2010). Frail patients, like all patients, have the right to information, participation, and tools for decision-making, as well as the ability to choose their treatment. The informants in this study belong to the frail older patient group—without cognitive disorders, however.

The findings of this study indicate experienced and perceived shortcomings in relation to information given during hospital stays. The shortcomings were related to insufficient information about care and medication, also given hastily in noisy surroundings. Patients perceived they were sent home too early, without a discharge call, and too many healthcare professionals were involved without continuity. Moreover, the patients expressed a need for more healthcare services, as they feel an inability to cope with the daily situation at home, they had no one to talk to, not being content with their lives. 

The patients described situations when they did not understand or perceive changes in their medication, such as drugs being prescribed or withdrawn, which led to different kinds of problems. Treatment with drugs is an important part within frail older patient care, and individual patient-adjusted information might lead to safer medication for these patients [15]. According to some informants in this study, too many people were involved, and the information was given too quickly or at an inappropriate time. The majority of the informants in this study also experienced and perceived varying degrees of dissatisfaction with their hospital discharges. Hansson et al. [6] has confirmed that healthcare professionals also claim that coordinated planning for frail elderly people is often lacking. Some of the informants could not recall participating in any discharge call at all, whereas others said it had been too brief. This perception may partly be explained by the noisy environment, confirmed by the authors’ observations, that frail older patients found themselves in during their hospital stay. The context in which the patients were sometimes given information contained elements of disturbing sounds, ringing telephones, visiting relatives, and wall-painters trying to do their work in the middle of the medical ward. The decision-making routine within Scandinavian hospitals’ so called “medical rounds” could also explain some informant dissatisfaction concerning received information. In this routine, nurses and doctors gather around the patient’s bed and decide together whether the patient should be discharged or needs to stay for further examinations or continuous care.

This study contributes to increasing knowledge in healthcare professionals’ communication with frail older persons. There were frail older persons, as cognitively healthy, not always feel well-informed, so the professionals’ communication with them can be said to be insufficient. Thus, this study confirms extant research; however, this study provides more details about the value of information and participation for frail older persons’ sense of well-being after discharge from hospital, also. To have frail older persons feel safe at home can decrease the need for readmission to hospital.

Health literacy presupposes that information is given to the patients. As the patients in this study were cognitively healthy and almost ready to be discharged from the hospital, it could be supposed that they have the necessary cognitive capacity to access, understand, and appraise any health information that is provided [22]. However, a non-optimal environment surrounding the frail older patients might lead to reduced health and information literacy. Sykes et al. [20] also include the context in the definition of health literacy, and this study confirms the importance of calm surroundings when communicating with patients. But if the hospital fails to give the patients sufficient information, it does not matter how good the patient’s own capacity of health literacy is. 

The concept of health literacy can be seen as a type of broader decision-making, which can be improved by political governance in order to provide more equal terms within healthcare [20]. In order for frail older persons to be able to understand and consider important decisions regarding their own healthcare, professionals should adjust the environment in which this care is performed and allow more time for informal talk with, and medical informing for, these patients. Seniors with adequate health literacy also need more motivational information to participate in medical decisions, since they constitute a patient group which is less inclined to adhere to discharge instructions [40]. It is also important to maintain continuity [16]. This study has not provided any findings supporting the frail older people’s participation in medical decisions. However, person-centered care (PPC) has been shown to increase patients’ knowledge and to contribute to increased patient participation [27,41]. There should be a routine for both informing patients about their important role in decision-making and for giving patients tools for understanding the consequences of their choices [42]. 

Moreover, PCC is intended to create a relationship between healthcare professionals and the patient as partners and equals. If PCC is not fully implemented, the frail older patient cannot participate in their own healthcare [29]. Thus, health literacy will not emerge when the frail older patient is not fully informed. Those two different perspectives, health literacy and PCC, could complement one another. PCC can provide better conditions to give information in a health literacy-friendly way, which makes it easier for frail older persons to participate in a partnership in care. 

Findings in this study also emphasize the environmental aspects of health literacy combined with PCC, considering the importance also of the explicit arrangement of the context and environment where the communication with frail older persons takes place. However, this study has not emphasized how hospital professionals handle deficits in health literacy of patients and how they could increase PCC, as discharge calls are not observed. Health literacy can be improved by using peaceful and quiet environments without too many distractions when informing frail older persons, as this patient group requires such a context in order to understand information about their own health and to participate in their own care. The patients in this study who were given the opportunity to participate and who were treated as partners within person-centered care felt both satisfied with their discharge call and well-informed about their received care and medical treatment. PCC, which involves shared decision-making, aids decision [41] and meaningful encounters [43], gives frail older persons the possibility to confirm and retain a position in the social world, and increases their well-being and independence [10].

The recollection of the frail older persons‘ memory can be noticed according to the study’s validity and reliability, as we have not collected any data on their exposure to anticholinergic and sedative medications that could cause poorer cognitive functioning [44]. This fact might have had an effect on the validity and reliability of the results. A limitation of this study is that the informant group was fairly small. The context and setting of the interviews might also be problematic, since disturbing events that took place on the ward, such as telephone calls and the fact that rooms designed for four patients were usually used by five patients, might have caused misunderstandings. However, the fact that the interviews were conducted in the environment in which the patients were cared for gave the researchers a better understanding of the noisy environments that frail older patients experience during their hospital stays. Another limitation to this study is that the hospital professionals were not observed during the moment the information at discharge was given to the frail older patients. However, the interviewer is well acquainted with the hospital environment where the information is given, as an experienced nurse.

## 5. Future Research

The medical round routine might work well for the professionals involved, but the findings of this study point out whether this arrangement suits frail older patients. Further research could possibly, in greater detail, examine the frail older patients’ experiences of their hospital care and how medicals rounds could be arranged in order to increase the patients’ health literacy.

The implementation of PCC is also of vital importance. However, the use of the concept of health literacy and PCC in combination does not always emphasize the explicit arrangement of the context and environment where the communication with the frail older person takes place. Therefore, more studies should be conducted in order to consider the importance of the arrangements around the sessions where healthcare professionals are communicating with the frail older person.

## 6. Conclusions

The frail older patients, however cognitively healthy, experienced receiving insufficient information about their care and rehabilitation, or receiving such information in noisy surroundings. They experienced situations of misunderstanding related to their medication, which indicates the need for appropriate discharge calls for frail older patients according to the person-centered care (PCC) approach. The frail older patients expressed feelings of distress concerning the future, caused by hasty admissions or relatives’ problems to handle the situation. It could be difficult to obtain health literacy for frail older persons in such a context where information is given, even though the frail older persons have their own capacity to understand, appraise, and apply health information in order to make judgments and take decisions regarding their care and everyday life. 

## Figures and Tables

**Table 1:** Examples of meaningful units, condensed meanings, and codes.

Meaningful Units	Condensed Meanings	Codes
”I got information about the discharge, but it was done very hastily and unexpectedly late on a Sunday evening… they had a lot of work to do… so I didn´t remember much of what they said”	”The information about discharge was given too hastily for me, I could not remember what they said”	Hastily information at discharge
